# The Pharmacist Prescriber: A Psychological Perspective on Complex Conversations about Medicines: Introducing Relational Prescribing and Open Dialogue in Physical Health

**DOI:** 10.3390/pharmacy11020062

**Published:** 2023-03-22

**Authors:** David Rogalski, Nina Barnett, Amanda Bueno de Mesquita, Barry Jubraj

**Affiliations:** 1 Camden and Islington NHS Foundation Trust, London RG24 9NA, UK; 2 London North West University Healthcare NHS Trust, London HA1 3UJ, UK; 3 Medicines Use and Safety Division, NHS Specialist Pharmacy Service, London HA1 3UJ, UK; 4 UKCP Systemic, CNWL (Central Northwest London), London EC3N 2LU, UK; 5 School of Pharmacy, University College London, London WC1E 6BT, UK

**Keywords:** relational prescribing, psychodynamic, open dialogue, pharmacist, person-centred care, shared decision making, alliance, medication, placebo, overprescribing, adherence, psychodynamic psychopharmacology

## Abstract

Pharmacists have traditionally supported the prescribing process, arguably in reactive or corrective roles. The advent of pharmacist prescribing in 2004 represented a major shift in practice, leading to greater responsibility for making clinical decisions with and for patients. Prescribing rights require pharmacists to take a more prescriptive role that will allow them to contribute to long-standing prescribing challenges such as poor medication adherence, overprescribing, and the need for shared decision-making and person-centered care. Central to these endeavors are the development and possession of effective consultation skills. University schools of pharmacists in the UK now routinely include consultation skills training, which is also provided by national education bodies. These challenges remain difficult to overcome, even though it is understood, for example, that increasing the effectiveness of adherence interventions may have a far greater impact on the health of the population than any improvement in specific medical treatments. More recently, a concerted effort has been made to tackle overprescribing and the harm that may occur through the inappropriate use of medication. In routine pharmacy work, these priorities may linger at the bottom of the list due to the busy and complex nature of the work. Solutions to these problems of adherence, optimizing benefits of medication, and overprescribing have typically been pragmatic and structured. However, an arguably reductionist approach to implementation fails to address the complex patient interactions around prescribing and taking medication, and the heterogeneity of the patient’s experience, leaving the answers elusive. We suggest that it is essential to explore how person-centered care is perceived and to emphasize the relational aspects of clinical consultations. The development of routine pharmacist prescribing demands building on the core values of person-centered care and shared decision making by introducing the concepts of “relational prescribing” and “open dialogue” to cultivate an essential pharmacotherapeutic alliance to deliver concrete positive patient outcomes. We provide a vignette of how a clinical case can be approached using principles of relational prescribing and open dialogue. We believe these are solutions that are not additional tasks but must be embedded into pharmacy practice. This will improve professional satisfaction and resilience, and encourage curiosity and creativity, particularly with the advent of all pharmacists in Great Britain becoming prescribers at graduation from 2026.

## 1. Introduction

In the UK, pharmacists have developed clinical roles—for example, hospital-based specialists and those working in general practice. Over the last nearly 20 years, pharmacists have had the opportunity to undertake an additional qualification to train as non-medical prescribers who can then operate in a variety of settings. Conversations about medicines are undertaken by a range of health professionals, including doctors, nurses, pharmacists, and pharmacy technicians. These are often transactional, focusing on the practicalities of the what, why, and when to use medicines safely and effectively [1]. As our understanding of medicines use has grown and pharmacy has become increasingly embedded into general practice (in the UK pharmacy staff are working alongside doctors and nurses in this setting) and patient-facing hospital services, deeper conversations with patients are happening about their motivations, beliefs, and fears, often in relation to their life circumstances, which impact their use of medicines. As such, the dynamics of prescribing are complex, and the relational aspect forms a large part of the therapeutic outcome [2,3,4]. Health outcomes can be improved through clinical conversations, where the clinician is “present” to improve engagement, trust, and rapport. The clinician must also seek to understand the wider impact of medicine taking and the consequences of diseases. Previous articles have highlighted the importance of clinical empathy, defined as appropriate empathy demonstrated in a clinical setting, in medicine optimization [5]. Clinical empathy allows pharmacists to engage patients in consultations about their thoughts and feelings around medication to identify ongoing pharmaceutical problems and to help them get the most from their medicines. This article furthers the thinking not on “what” to prescribe but on “how” to prescribe, providing a relational framework to get the best outcomes for patients, improve prescriber resilience, and support clinicians’ development.

“By far the most frequently used drug in general practice was the doctor himself, i.e., it was not only the bottle of medicine or the box of pills that mattered, but the way the doctor gave them to his patient—in fact the whole atmosphere in which the drug was given and taken.” [6].

## 2. Meaning and Medication: The Evidence Base

Medicine taking is a complex human behavior. There is growing evidence that non-pharmacological factors play an active role in treatment outcomes [7]. For example, well-known factors are the placebo and nocebo effects, where a patient’s expectations of treatment and interaction with the clinician significantly determine how they will experience the intervention. The placebo effect is a phenomenon in which some people experience a benefit after the administration of an inactive lookalike substance or treatment. The nocebo effect, the converse of the placebo response, occurs when patients who harbor expectations of harm are more likely to respond adversely to medications [8]. Placebo responses are real effects; for example, we know that blood pressure is reduced in placebo trials [9]. In relational prescribing, we focus on the clinician–patient dyad and make use of the therapeutic relationship as a healing agent. By “relational”, we mean external relationships, i.e., the clinician–patient interaction, family interactions, and internal relationships, i.e., relational templates in the mind of the patient and the clinician. A relational framework supports a “whole person and whole life” perspective on human distress and addresses problems related to fragmentation of care [3,10].

Research has been undertaken into the psychosocial/relational aspects of prescribing. There is evidence that suggests improved adherence to medicines improves health outcomes, and that more effective conversations will contribute to these improved outcomes [11,12,13,14]. A link between communication and patient adherence has been observed extensively in general medicine. A meta-analysis synthesizing results from correlational and experimental studies found the odds of a patient adhering to be 2.16 times greater if their doctor is a good communicator [15]. Several studies and reviews clearly showed a correlation between effective communication and improved health outcomes [9]. The outcomes affected were emotional health, resolution of symptoms, function, pain control, and physiological measures such as blood pressure and blood sugar concentration. Other studies have shown less organ damage among patients with systemic lupus erythematosus [16] and higher quality of life among breast cancer patients [17].

However, it is suggested that doctors tend to overestimate their abilities in communication. Tongue et al. [18] reported that 75% of the orthopedic surgeons surveyed believed that they communicated satisfactorily with their patients, but only 21% of their patients reported satisfactory communication. Surveys have consistently shown that patients desire better communication with their doctors [19]. Kaplan et al. showed that patients tended to leave doctors who failed to involve them in decisions [20]. In this observational study of 7730 patients and their doctors, a third of those rating doctors in the lowest participatory quartile changed doctors the following year. Under pressure to contain costs, doctors respond by increasing their practice volume, meaning a corresponding decrease in time spent per patient [21]. This is a false economy if, as Kaplan suggests, it results in patients abandoning that doctor. There are many barriers to good communication in the doctor–patient relationship, including patients’ anxiety and fear, doctors’ burden of work, fear of litigation, fear of physical or verbal abuse, and unrealistic patient expectations [22]. A good clinician–patient relationship can increase job satisfaction and reinforce patients’ self-confidence, motivation, and positive view of their health status, which may influence their health outcomes [23,24].

A range of tools and models for assessing patients’ perspectives of illness and treatment have been developed and can be helpful—e.g., the Beliefs about Medicines Questionnaire (BMQ) [25] and the Medication Adherence Report (MARS). There are frameworks for understanding treatment-related behaviors with a particular focus on adherence to medication—e.g., the Necessity-Concerns Framework and Perceptions and Practicalities approach [26]. Clinicians often approach their work with a set of templates and internal algorithms that help them make decisions about how to respond. Unfortunately, one of the consequences of this is that patients and carers can be left feeling unheard. While there is interest in exploring a more person-centered, psychological approach to pharmacy consultations using behavior-changing techniques [27,28], there are barriers to a person-centered approach that need to be addressed [29]. A conversation can too often be about extracting or imparting information (“doing to”), rather than “being with” the patient and whatever is happening in the present. Finally, these tools can help clinicians in “being with” the patient to deliver the optimum care and think about perceptual barriers to adherence (e.g., taking medication means you are weak or is a reminder of being unwell) and practical barriers to adherence (e.g., forgetfulness or regimen complexity) [5].

It is important to note that a substantial body of research exists from prescribing centers around the practices of doctors. We contend that their typical findings are applicable to pharmacists, though there are some distinctions. For example, as prescribing is a more recent pharmacy activity, it would be expected that consultation skills will take time to develop [30].

Our two proposed approaches for incorporation into clinical practice are now described, starting with relational prescribing.

### 2.1. Relational Prescribing

**Figure 1:**
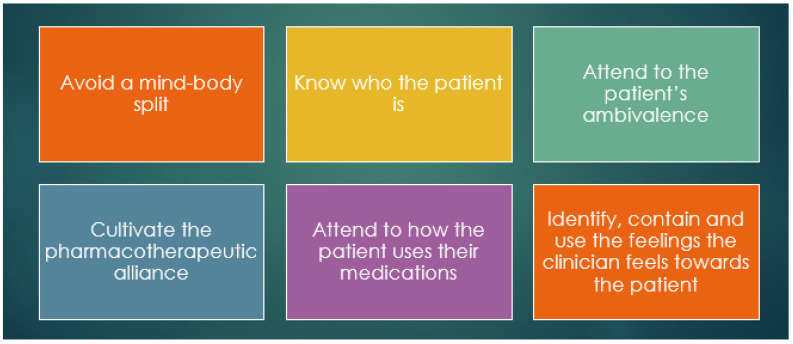
Overarching principles to relational prescribing (principles adapted from [31].

The principles of relational prescribing are described below (Figure 1).

In this article, we use the term “relational prescribing”, but the principles are based on the framework for psychodynamic psychopharmacology. We describe here the principles of relational prescribing in more detail.

#### 2.1.1. Avoid Mind–Body Split

There is mounting evidence that the mind and body are linked [32]. It is important to recognize that illness and responses to treatment always represent a complex interplay of biological, psychological, and social factors. Avoiding a mind–body split in approaching the patient may sound straightforward, but in practice it requires a great deal of vigilance and discipline. Clinicians face numerous pressures to approach the patient reductively. These pressures find their roots in Western culture and language, a consequence of Cartesian dualism that has been deeply ingrained [31]. Cartesian dualism is the view that the mind and body are distinct and separable.

#### 2.1.2. Know Who the Patient Is

Who the patient is and how they relate to themselves and those around them greatly affect the outcomes and expectations of treatment. Attachment and psychodynamic theory provide us with helpful ways to understand how people develop and form relationships. While it can be helpful to have a developmental history, this is typically not possible with a limited time for consultation, or is not directly accessible. However, it is possible to consider the patient’s broader developmental aims and explore the relationship to treatment. Questions about who the patient is and their deeper motivations can re-orient the conversation to explore aims of the treatment in ways that go far beyond mere symptom-reduction.

“It is much more important to know what sort of a patient has a disease than to know what sort of a disease a patient has.” (attributed to Sir William Osler, cited in [33])

#### 2.1.3. Attend to the Patient’s Ambivalence

Among patients with chronic illness, approximately 50% do not take medications as prescribed [34]. This ambivalence is no surprise, given the essentially ambivalent nature of medicine. Ambivalence is a pervasive characteristic of mental life whereby contradictory feelings and impulses coexist. The word “pharmacy” finds its derivation in the Greek word “pharmakon”, which contains the dual meanings of cure and poison. Even if medications do not carry a significant side-effect burden, they typically represent a burden. At the very least, medications pose the burdens of time, mental space, and often money. There may be ambivalence towards change, the illness itself, the prescriber, or loss of symptoms. For example, there may be a wish not to think of oneself as having an illness because of the distress it might bring. Taking a medication could feel like an intolerable admission of weakness or personal failure.

#### 2.1.4. Cultivate a Pharmacotherapeutic Alliance

In addition to ambivalence about medications and about illness, patients may also be ambivalent about their clinicians. Although every pharmacy student should know that the clinician–patient relationship is of central importance in the practice of medicine, it typically receives far less attention than the more “specific” treatments offered. There is a need not only to gain the patient’s respect through a combination of competence, warmth, presence, tact, and empathy, but also to respect the patient’s capacities as a participant in the therapeutic endeavor [4,35].

#### 2.1.5. Attend to How the Patient Uses Their Medication

Conscious thought represents a compromise between competing wishes, impulses, demands, and prohibitions. The clinician will have to come to terms with a certain degree of irrationality in the patient’s use of medication by respecting those competing agendas in the patient. In such cases, not much needs be done, except to cast some light on the irrationality. However, there are times when patient’s medications can be turned to serve some serious countertherapeutic end which requires intervention—for example, intentional poor adherence.

#### 2.1.6. Identify, Contain, and Use Feelings the Clinician Feels towards the Patient

It is not just knowledge and competence that determine clinical decision making [36]. Other clinician-related factors also determine how quickly medication is prescribed. Prescribing under pressure is an important topic in social pharmacology [37]. It is important to recognize when we do things outside of our usual practice. Sometimes, we are alerted to this when patients deteriorate significantly. Strong feelings evoked by patients, either positive or negative, are another signal to pause and consider whether one’s clinical judgment is affected. It is important to understand what emotions (Box 1) are also evoked in us and what they might be telling us about the clinical interaction. Without this awareness, atypical dosages and complex and/or irrational medication regimens may develop in response. The feelings raised can be used to strengthen the pharmacotherapeutic alliance.

A relational way of working does not require a complicated formula. It may mean that instead of having symptom-focused goals, one asks about broader developmental goals, thereby reflecting on and considering medications in the service of these goals.

Box 1 **Feelings aroused in clinicians.**

▶ Empathy
▶ Warmth
▶ Caring/Helpful/loving/competence
▶ Frustration/anger
▶ Helplessness/despair
▶ Anxiety/sense of persecution/incompetence
▶ Shame
▶ Repulsion/disgust

### 2.2. In Summary: Relational Prescribing

Relational prescribing adds richness and meaning to the complex processes that occur in the clinician–patient dyad to help improve health outcomes, and it makes use of the therapeutic relationship as a healing agent.

## 3. Open Dialogue

Open dialogue is a distinct but additional area of focus inviting people who are important to the patient into the clinical space to make use of the relationships. Medications can often become the focal point of the MDT meetings; and any changes without involving the patient can lead to them feeling dehumanized and “talked about” rather than “talked with.” The realities of iatrogenic harm and well-documented physical responses to withdrawal from some medications are now gaining much more attention [38].

This presents a significant opportunity for prescribing, where dialogue becomes the main and primary element of good treatment. This means that creating context rather than diagnosis is our baseline.

Open dialogue is a community-based approach to mental health care which, at its heart, is informed by family and systemic practice. Open dialogue is applicable across healthcare settings [39]. A premium is placed on establishing connections between clinicians and patients, and between the patient and his or her social network. It was developed over a 30-year period in Western Lapland, Finland to tackle their entrenched problems of overuse of hospital beds and medications, and covering a large area with few resources. The research on the population receiving this approach showed radically better outcomes [40,41]. Ongoing studies are occurring to see if these results can be replicated in large urban areas such as London and for a range of mental health conditions [42].

In this approach, from first contact, every meeting becomes an opportunity to have a “therapeutic” conversation. In the mental health arena, open dialogue seeks to recruit and upskill the people around someone to enable them to be part of understanding and solving that person’s mental health problems [43]. Instead of asking, “What is wrong with you?”, open dialogue encourages a clinician to first ask, “Who is important to you?” Clinicians should always operate as a team instead of on their own and work with the service user’s self-defined social network. Open dialogue has radical transparency at the core of its approach throughout, adhering to the mantra “nothing about us without us”.

Open dialogue provides a reorientation of staff priorities and rigorous training in specific skills to focus on the development of trusting relationships, and most vitally, compassionate, responsive listening. It moves an organization away from a micro-managed, “high fear culture” towards a “high trust culture”, where clinicians are respected for their skills and individual service users do not have the content of their lives discussed disrespectfully away from their presence.

Open dialogue is a moral approach ensuring that patients feel heard, included, and respected. In 2021, the World Health Organisation published its guidance on “Community Mental Health Services: Promoting Person-Centred and Rights Based Approaches”. Within this guidance, open dialogue is cited as a best-practice example.

Open dialogue is being introduced across the UK in the mental health setting, and there are key open dialogue principles that create a framework of safety that could allow for conversations to occur prior to medications and then if needed. Help with open dialogue is immediate. It includes a flexible and mobile network around a person, with consistent staff and an open-door policy. This creates a framework of responsibility that is proven as safer for the patient. As prescriptions need to be clinically appropriate and regularly monitored, being able to offer structured, openly discussed medication reviews in an open dialogue network meeting feels like natural synergy. We believe there is a place for the principles of open dialogue in the general medical setting. Pharmacists within network meetings can address medical optimization by understanding connections between a patient’s physical and mental health, but equally vitally, between a patient’s family and network.

We cannot prescribe medicines in silo or have individualistic solutions to systemic issues. Responses are based in context and a person’s system. We therefore need systemic integrated solutions with relationships and authentic connections at the heart of all decision making. This may take more time, as a therapeutic alliance does take time, but it is an investment that will pay back, as people will feel heard.

## 4. Shared Decision Making and Coaching

Shared decision making is a collaborative process where clinicians and patients work together to help patients make decisions that are right for them [44]. The process combines the clinician’s expertise in treatment options, risks, benefits, and evidence, together with what matters to the patient—their preferences, goals, beliefs, and values [45]. Many clinicians believe that shared decision making only happens with equal participation between both parties, but as many clinicians will know, patients vary in the amount and type of input they want in decisions. We believe the focus of shared decision making should be on building an alliance with patients, taking account of cultural contexts and preferences, to deliver the collaborative conversations in a way that makes sense for each individual patient. In the context of open dialogue, we suggest some of these elements of what is known in England as a “structured medication review” could be incorporated into open dialogue conversations.

A coaching approach to consultations [46] is a helpful framework in which embed shared decision making, through identifying, at the start of a consultation, both a shared agenda and shared goals. In this way, both patient and clinician are clear about what they want to get out of the consultation and can check, at the end, that they have achieved what was needed. From a prescribing perspective, this is an effective way to undertake a structured medication review. The person-centered polypharmacy process [47] ensures that the patient and clinician discuss the issues they both want to bring to the consultation around medicines (step 3, identify medicines with potential risks; step 4, assess risks and benefits in the context of the individual patient). This also encourages the patient to raise issues, whether around physical or mental health, and the clinician to consider the impacts of the medication on the patient’s overall situation, physically and mentally. The recent National Overprescribing Review Report in England [48] highlighted the importance of including non-pharmacological options in these conversations. It is also important to ensure that patients have time to reflect on options following the discussion, and to offer additional information, signposted according to the patient’s needs.

In order to be effective, we suggest that there needs to be a focus on the relational element of structured-medication-review conversations, where the pharmacist is “emotionally available” to the patient, and “expertly available”. Pharmacists are well-trained to identify practical issues which support patients to use and take medication safely and effectively, and these have traditionally been undertaken in community-pharmacy or hospital-ward settings. Evidence is emerging around the effectiveness of pharmacy consultations, ranging from practice-based audits that explore the severity of conditions with which patients are presenting in community pharmacy [49], to the use of process measures that explore the value of hospital-ward-based consultations [50]. As the role of the pharmacist has expanded in the UK, pharmacists are now conducting consultations in general-practice clinics, hospital clinics, and community pharmacies’ consultation rooms. An increasing number of pharmacists are now prescribers, and from 2026, all pharmacists in Great Britain will be able to prescribe following successful completion of their undergraduate degree. The effectiveness of pharmacy consultations is being researched and reported with increasing frequency in the literature [51]. The need for more in-depth conversations with patients to develop relational prescribing is particularly timely in this context.

The following vignette illustrates a single medication-review consultation which moved from transactional to relational to open dialogue in general health. The aim is to demonstrate the creativity and meaning these frameworks bring.

## 5. Moving Forward—The Cultural and Educational Imperatives

To realize the benefits of relational prescribing and open dialogue, the change in culture that has begun with a greater emphasis on person-centered care and shared decision-making must gather pace. Patient feedback, role modelling, and clinical supervision [52] can be used to support changes in consultation practice. Moreover, with the graduation of pharmacist prescribers being scheduled for 2026, the educational imperative is clear. Appropriate and targeted undergraduate education, foundational training, support in the early years, and ongoing professional development, feeding into revalidation, will need to embed these concepts to ensure that they become business as usual for future generations of pharmacist prescribers.

## 6. Vignette

Luigi is a man in his mid-50s who has type two diabetes and is otherwise well. His wife asked him to speak to the pharmacist about his prescribed statin medication because he had not started taking it. He said she was scared he would have a stroke.

Our conversation began with discussing what he knew about why had been prescribed medication. He said that he had been told that he had high cholesterol, and the prescriber had made him aware that this medication would be required for the rest of his life. He said he would try to manage this by increasing his exercise, reducing dietary cholesterol, and taking plant-sterol-containing drinks every day. When, three months later, he had blood tests, he looked disappointed to learn that his cholesterol level had only decreased by a small amount. We then discussed how much he expected his cholesterol to have fallen and discovered that he thought that diet and exercise would allow his cholesterol to return to the normal range.

Transactional: I explained that there was a limit to how much we can control our cholesterol in this way. For some people, medication is the only way to reduce it enough to make a significant difference, and he was reassured by this.

*Armed with this information and the medication, would he now be happy to start taking it? What further discussion might be of benefit?*

Relational prescribing: I said that it seemed like he had tried very hard to take control of his health and asked how it felt that his cholesterol was still not in the normal range. He said it had felt like a failure not to have been able to reduce his cholesterol with diet and exercise. He had tried to manage it by swimming three times a week, reducing fats in his diet, using plant-sterol spread, and taking a plant-sterol-containing drink every day. He said that he thought he could do it because he had been successful at avoiding the need for medication for his type two diabetes through diet and exercise.

I acknowledged how disheartened he was with his situation. I then explained that there was a limit to how much we can control our cholesterol in this way. For some people, medication is the only way to reduce it enough to make a significant difference, and he told me he felt reassured now that he understood the same was not true for cholesterol.

Then I asked him about the conversation he had when statins were first prescribed. He told me that the prescriber had made him aware that this medication would be required for the rest of his life. I asked about how this made him feel, and he told me that it had triggered thoughts of aging and mortality. He said it made him feel old. I listened to him and understood that, for him, “medication for life” meant “medication until death”, raising frightening feelings around vulnerability and death. I recognized feelings in myself about the meaning of “medicines for life” and the fear it could bring.

To try and enhance his sense of autonomy (control over his health and cholesterol) and hopefulness (for a healthy future) I explained that rather than considering any medication “for life,” we now view all medication as needing regular review to ensure it continues to benefit a patient at that time. I asked what he thought about this, and he told me that the idea of regular review made sense to him.

We then talked about the idea of him taking the statin for a short time, reviewing how it worked for him, and then deciding if it would be appropriate to continue, stop, or change.

He agreed to take a statin for three months, while he continued to use diet and exercise as before; then he would organize a cholesterol test with his GP and return for an appointment with me to discuss it. He seemed happy that we had come up with a manageable, collaborative, and comfortable way forward.

Using the relational framework below breaks down the interaction in more conceptual detail.

### 6.1. Avoid Mind–Body Split

His mind is concerned about his mortality and advancing years while there is a more immediate threat to his body, one that is outside his control.

### 6.2. Know Who the Patient Is

He is a middle-aged married man who perhaps struggles with the more vulnerable aspects of himself, requiring support from those close to him to nudge him to engage with help. He has already had a significant threat to his health in the form of diabetes, through which he has been able to take control of his health, but it has been different this time. He is resourceful and has healthy engagement with non-pharmacological interventions.

### 6.3. Attend to the Patient’s Ambivalence

He was worried about what he is able to control about his health and about needing assistance. This brought up strong feelings about his mortality. Knowing that this is important to him, I provided him with his own autonomy and framed the treatment as taking control of his care and looking after himself for the future. There is also the opportunity to revisit the need for treatment, making it feel more digestible. The patient may see the first prescriber as being rigid, or not know how much this experience has impacted him. Further exploration allowed for a way forward.

### 6.4. Cultivate a Pharmacotherapeutic Alliance

He felt heard and able to share his thoughts. He was transparent about failing to address the problem himself, and the pharmacy professional was transparent about the need for medication. He also responded well to the idea of support that was offered for the forward journey of treatment.

### 6.5. Attend to How the Patient Uses Their Medication

The patient may return at times to the idea of not needing medication, and this will need to be kept in the mind of the professional to reinstall the boundaries using the information at hand, evidence, and best practices.

### 6.6. Identify, Contain, and Use Feelings the Clinician Feels towards the Patient

The pharmacist felt moved by the patient sharing his experience of what taking the medication meant to him. The uncertainty could be contained between them, and a way to move forward could be navigated.

### 6.7. An Open Dialogue Approach

With an open dialogue consultation, this patient would be asked who is important to him and that they could be invited into the consultation. In this vignette, the pharmacist may consider that it could be helpful to ask the patient if he would be comfortable with his wife joining the consultation. This is because via a relational lens and open dialogue, there is recognition that if one member of the family is unwell or takes medications. There is invariably an impact on other people, be that physical side-effects of medication or the patient and loved ones’ emotional responses to the medications.

As it was the patient’s wife who requested that he speak to the pharmacist, the pharmacist can hypothesize that this conversation between them may be difficult. The wife requesting that her husband speak to the pharmacist indicates a potential emotive topic that may benefit from exploration.

Open dialogue encourages the pharmacist to speak to and recognize his or her own feelings in the room. The pharmacist is not “doing something to” the patient but is taking the perspective of “working with”. The pharmacist is an expert in medicines but not an expert on the patients’ body, mind, family history, and dynamics. Therefore, the approaches used in this conversation are collaboration and exploration.

As the pharmacist recognized in himself feelings surrounding the idea of “medicines for life”, open dialogue would allow the pharmacist to openly express and acknowledge his own responses.

Pharmacist: “Something in me feels that the idea of taking a statin for life may feel uncomfortable for you?”.

Patient: “Yes, I feel like an old man and a failure as I really thought that my diet and exercise could sort out my cholesterol, I don’t want to take pills till I die”.

Pharmacist to wife: “I know you initiated this consultation, have you got concerns you want to share?”.

Wife: “Well, I know he probably needs them, but my brother died from a stroke at 53 whilst taking statins, so I feel really conflicted as the doctor said that once on them, he will never be able to stop them”.

*The wife may then cry and reach out to hold her husband’s hand.*

The pharmacist is moved in the light of this additional knowledge. “I can feel why this may feel frightening, how recent was this and what was your brother’s name?”.

His wife looks sad but pleased she has been “heard.” “His name was Robert, and it was only two years ago, he was also diabetic, and I can’t face another loss”.

The patient then consoles his wife and holds her hand, and this moment seems to bring them into a collaborative space to think openly alongside the pharmacist.

Pharmacist “If Robert were here with us, what do you think he may say about starting the statins?” *(This question acknowledges that the wife has been truly heard, invites Robert into the conversation, creating another “voice” and recognizing that someone who has passed can still be very present in their loved one’s minds and hearts. In this case, Robert is very present in this decision, so the pharmacist is taking a relational yet processed risk).*

Wife: “Robert may say start them but remain vigilant regarding exercise and diet”.

Pharmacist to both: “The evidence is that cholesterol doesn’t always respond to lifestyle changes and often needs statins”.

Patient to wife and pharmacist: “I know she [looking at wife] is concerned, and I appreciate how hard she… well we all took Robert’s death at such a young age”.

Wife: “Yes, it has been and still remains hard, Robert left two teenage girls and our kids miss their uncle and they are very close to their cousins”.

Pharmacist (with a better understanding of the context): “Medicines do not need to be taken with a view to never stopping, would it feel comfortable to start for twelve weeks, and then return for a review so that we can then think together again?”.

Patient: “So you’d monitor me that regularly?”.

Wife: “And could we all meet together again for the review?”.

Pharmacist: “Yes of course, and we can then check how you are responding and do continue the exercise and diet regime”.

## 7. Summary

Pharmacy education, training, and development have begun to emphasize the importance of effective clinical consultations and the nature of the pharmacist–patient relationship. Effective, person-centered consultations, with an emphasis on shared decision making and an empathic approach, are accepted as a necessary underpinning to the appropriate use of medicines. The principles of relational prescribing, open dialogue, and shared decision making in the context of a person-centered consultation, present an opportunity for a mindset change amongst clinicians. Pharmacy education has traditionally emphasized knowledge and skills amongst the traditional factors of learning. As curricula develop, existing person-centered communication tools and shared decision-making processes can be embedded with an emphasis on values and behaviors of the clinician. There needs to be a focus on supervision and training to help pharmacists engage in these approaches, which can feel daunting. Relational prescribing and open dialogue are powerful aids to developing these attributes of future pharmacists.

## Data Availability

Data sharing not applicable.
